# P-821. Invasive Streptococcus pyogenes Infections: Is Anti-toxin Therapy Necessary?

**DOI:** 10.1093/ofid/ofae631.1013

**Published:** 2025-01-29

**Authors:** Daniel Kinsey, Megha Jagannathan, Tamara Jordan, Rachel M Kenney, Michael Veve, Anita Shallal, Geehan Suleyman

**Affiliations:** Henry Ford Hospital, Detroit, Michigan; Henry Ford Hospital, Detroit, Michigan; Henry Ford Hospital, Detroit, Michigan; Henry Ford Hospital, Detroit, Michigan; Henry Ford Health, Detroit, Michigan; Henry Ford Health, Detroit, Michigan; Henry Ford Health, Detroit, Michigan

## Abstract

**Background:**

Invasive *Streptococcus pyogenes* or group A Streptococcus (GAS) carries a high morbidity and mortality rate. In addition to penicillin, adjunct anti-toxin therapy (AT) with linezolid or clindamycin is the standard of care. Although the use of AT is supported by *in vitro* data and observational studies, there are limited conclusive data supporting its efficacy in reducing GAS mortality. Our study aimed to compare clinical outcomes of patients receiving AT versus those who did not.

**Figure:**
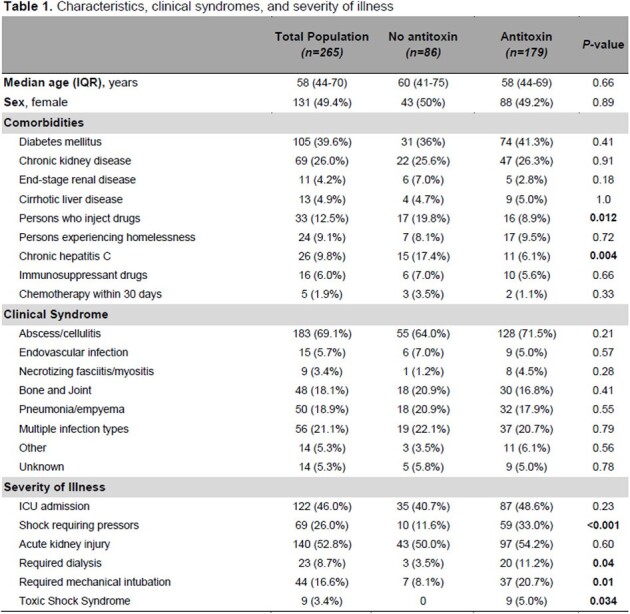

**Methods:**

This was a retrospective cohort study of hospitalized patients with positive blood cultures for GAS at our five-hospital system from June 2013 to August 2023. Patients were identified through Microsoft SQL Server. Patients who received AT therapy for > 48 hours were defined as the AT group; the control group did not receive AT therapy. Patients who received AT for < 48 hours were excluded. Collected variables included demographics, infection & microbiological characteristics, and clinical outcomes. Data was analyzed using SPSS.

**Figure:**
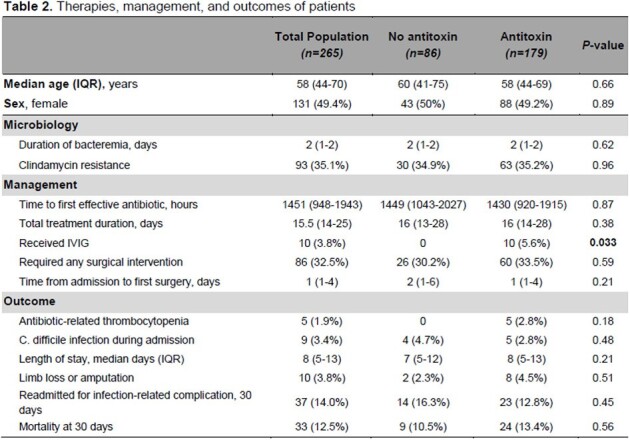

**Results:**

265 patients were included in the study of whom 179 (68%) received AT (Table 1). About half were female, and the median age was 58 years. Comorbidities were overall similar between the two groups. Persons who inject drugs or with chronic hepatitis C were more common in the control group. Abscess/cellulitis was the most common clinical syndrome in both groups. Shock requiring vasopressors, need for dialysis and mechanical ventilation, and toxic shock syndrome were prevalent in the AT group. There was no difference in the duration of bacteremia, clindamycin resistance, and management between the two groups except for receipt of intravenous immunoglobulin (IVIG), which was more common in the AT group (Table 2). Outcomes, including length of stay, readmission for infection-related complications, and 30-day mortality, were similar between the two groups.

**Conclusion:**

Utilization of AT for invasive GAS infections correlated with severity of illness, but clinical outcomes did not significantly differ between patients receiving AT and those who did not, suggesting a potential opportunity for antimicrobial stewardship. Further research is needed to determine whether AT should be reserved for patients with select infectious syndromes

**Disclosures:**

**Rachel M. Kenney, PharmD, BCIDP**, Medtronic Inc: Spouse is an employee, stockholder

